# Message from the President

**Published:** 2008-01

**Authors:** Niti Khunger

**Affiliations:** President, Association of Cutaneous Surgeons of India

Dear members,

These are exciting times for dermatology and dermatosurgery. Cutaneous and aesthetic surgery is beginning to play an increasingly important role in our day-to-day practice. New developments in this field continue to occur at regular intervals and the practicing physician is often bewildered by the scorching pace of technological advancement and the various options becoming available to treat patients. Under these circumstances, it is important not to lose one's objective viewpoint. Keep abreast of the latest advances, but decisions to reject older established procedures in favor of the new should be tempered by scientific evidence. It has been rightly said that not everything that is new is good, but everything that is good was once new.

It is the aim of the journal of our association to bring to you the developments occurring in the field of cutaneous and aesthetic surgery, laced with the experience and wisdom of the masters, and set against a scientific background. The journal will also serve as a platform for the exchange of ideas on the problems in dealing with different skin types, particularly ethnic skins, and hopes to fill the existing void in this area.

The official journal of our association (previously known as the Indian Journal of Dermatosurgery) has been reborn with a new look and new name. I profusely thank the editorial team for their efforts and labor in bringing out the journal. I also appreciate the support of our eminent advisors, national and international, and I am sure that with their guidance and with excellent teamwork, we will succeed in setting high standards for the journal.

I request you all to contribute your work, to give it your best; without that we cannot succeed.

**TEAM** stands for **T**ogether **E**veryone **A**chieves **M**ore!

